# Periodontal disease increases the host susceptibility to COVID-19 and its severity: a Mendelian randomization study

**DOI:** 10.1186/s12967-021-03198-2

**Published:** 2021-12-24

**Authors:** Yi Wang, Hui Deng, Yihuai Pan, Lijian Jin, Rongdang Hu, Yongyong Lu, Wenhai Deng, Weijian Sun, Chengshui Chen, Xian Shen, Xiu-Feng Huang

**Affiliations:** 1 School & Hospital of Stomatology, Wenzhou Medical University, Wenzhou, Zhejiang China; 2 Faculty of Dentistry, The University of Hong Kong, Hong Kong SAR, China; 3 Department of Urology, The First Affiliated Hospital of Wenzhou Medical University, Wenzhou, Zhejiang China; 4 Key Laboratory of Laboratory Medicine, Ministry of Education, School of Laboratory Medicine and Life Sciences, Wenzhou Medical University, Wenzhou, Zhejiang China; 5 Department of Gastrointestinal Surgery, The Second Affiliated Hospital of Wenzhou Medical University, Wenzhou, Zhejiang China; 6 Department of Pulmonary and Critical Care Medicine, The First Affiliated Hospital of Wenzhou Medical University, Wenzhou, Zhejiang China; 7 Key Laboratory of Interventional Pulmonology of Zhejiang Province, Wenzhou, Zhejiang China; 8 The Second Affiliated Hospital of Wenzhou Medical University, Wenzhou, Zhejiang China; 9 Wenzhou Medical University-Monash BDI Alliance in Clinical and Experimental Biomedicine, Wenzhou Medical University, Wenzhou, Zhejiang China

**Keywords:** COVID-19, Periodontal disease, Risk factor, Mendelian randomization

## Abstract

**Background:**

Emerging evidence shows that periodontal disease (PD) may increase the risk of Coronavirus disease 2019 (COVID-19) complications. Here, we undertook a two-sample Mendelian randomization (MR) study, and investigated for the first time the possible causal impact of PD on host susceptibility to COVID-19 and its severity.

**Methods:**

Summary statistics of COVID-19 susceptibility and severity were retrieved from the COVID-19 Host Genetics Initiative and used as outcomes. Single nucleotide polymorphisms associated with PD in Genome-wide association study were included as exposure. Inverse-variance weighted (IVW) method was employed as the main approach to analyze the causal relationships between PD and COVID-19. Three additional methods were adopted, allowing the existence of horizontal pleiotropy, including MR-Egger regression, weighted median and weighted mode methods. Comprehensive sensitivity analyses were also conducted for estimating the robustness of the identified associations.

**Results:**

The MR estimates showed that PD was significantly associated with significantly higher susceptibility to COVID-19 using IVW (OR = 1.024, *P* = 0.017, 95% CI 1.004–1.045) and weighted median method (OR = 1.029, *P* = 0.024, 95% CI 1.003–1.055). Furthermore, it revealed that PD was significantly linked to COVID-19 severity based on the comparison of hospitalization versus population controls (IVW, OR = 1.025, *P* = 0.039, 95% CI 1.001–1.049; weighted median, OR = 1.030, *P* = 0.027, 95% CI 1.003–1.058). No such association was observed in the cohort of highly severe cases confirmed versus those not hospitalized due to COVID-19.

**Conclusions:**

We provide evidence on the possible causality of PD accounting for the susceptibility and severity of COVID-19, highlighting the importance of oral/periodontal healthcare for general wellbeing during the pandemic and beyond.

## Introduction

Coronavirus disease 2019 (COVID-19), initiated by a novel severe acute respiratory syndrome coronavirus 2 (SARS-CoV-2), has become a global pandemic since March 2020. Despite the strict lockdown measures and implementation of vaccination, the number of COVID-19 cases and deaths has reached over 169 and 3 million respectively, according to the WHO report updated on 30 May 2021 [1]. Notably, the susceptibility and severity of COVID-19 are highly heterogeneous. Most individuals are presented with asymptomatic infection, while severe cases are developed into acute respiratory distress syndrome, multi-organ failure, and eventually death [2, 3]. Therefore, identifying potential risk factors for COVID-19 is of high priority.

Periodontal disease (PD), comprising gingivitis and periodontitis, is one of the most common immuno-inflammatory disorders that result from dysbiosis. Severe periodontitis was estimated to affect 11.2% of the general population, ranking the sixth most prevalent disease worldwide [4]. PD is characterized by the accumulation of multiple bacterial and subsequent destruction of tooth-supporting tissues, leading to tooth loss if left untreated [5]. Accumulating evidence from observational, intervention, and experimental studies has associated PD with a number of comorbidities, such as atherosclerosis, diabetes mellites, respiratory disease, cancer, etc [6, 7]. Moreover, our recent studies suggest that PD is a risk factor for myocardial dysfunction [8, 9]. The periodontal systemic associations could be briefly explained by the spillover of subgingival pathogens into the bloodstream, as well as a cascade of systemic inflammation and autoimmune damage from the host response [10].

Given the impact of PD on the systemic conditions, it is reasonable to assume that there is a possible linkage between PD and COVID-19. Indeed, several hypotheses have been made based on the previous evidence found in the shared risk factors (e.g., age, obesity, smoking) and bacterial co-infection [11, 12]. Moreover, the mucosal of the oral cavity could be a risky route for SARS-CoV-2 infection since it is highly enriched in the expression of angiotensin-converting enzyme II (ACE2), a major host receptor for SARS-CoV-2 [13]. It is recently demonstrated that patients with painful/bleeding gum are more vulnerable to mortality following COVID-19 infection [14]. Recent case–control studies further identify PD as a contributing factor for COVID-19 infection and complications [15, 16]. However, these observational studies are prone to confounders, and the causal impacts of PD on host susceptibility to COVID-19 and its severity have not been ascertained.

Mendelian randomization (MR) is a research method that examines the causal relationships between risk factors and disease outcomes by the use of genetic variants as natural experiments [17]. Its principle is comparable to randomized controlled trial (RCT). In RCT, participants are randomly assigned to experimental groups, while in MR, the randomization variable is genetic variants [18]. Although RCT is the gold standard approach to search for causal relationships, a well-designed RCT is expensive, time-consuming, and even impractical. Over the past decade, with Genome-Wide Association Study (GWAS) summary data being increasingly released for public availability, and developments of statistical approaches, MR analysis is becoming extremely valuable and increasingly applied in many important diseases. MR studies can confirm the associations reported by clinical observational studies, and reveal novel associations [17, 19]. Recently, multiple MR studies have been conducted to infer the causal associations of serval traits on the host susceptibility to COVID-19 and its severity, such as psychiatric disorders [20], glycemic traits [21], and cardiometabolic traits [22]. However, there has been no MR study investigating the potential causal relationships between PD and COVID-19.

Given the aforementioned observational evidence, we hypothesized that periodontal disease may accounts for an increased susceptibility and severity of COVID-19. To test our hypothesis, 2-sample MR analyses were performed to investigate the causal inference of genetically predicated periodontal disease on COVID-19 susceptibility and severity. Based on the framework of MR, genetic instruments robustly associated with PD-related Socransky phenotype were retrieved. This phenotype describes a high correlation and positive loading for periodontal pathogens [23]. COVID-19 infection and severe phenotypes, including hospitalized and severe cases from recently conducted GWAS, were included as the outcome of interest [24].

## Methods

### Study design

**Fig. 1:**
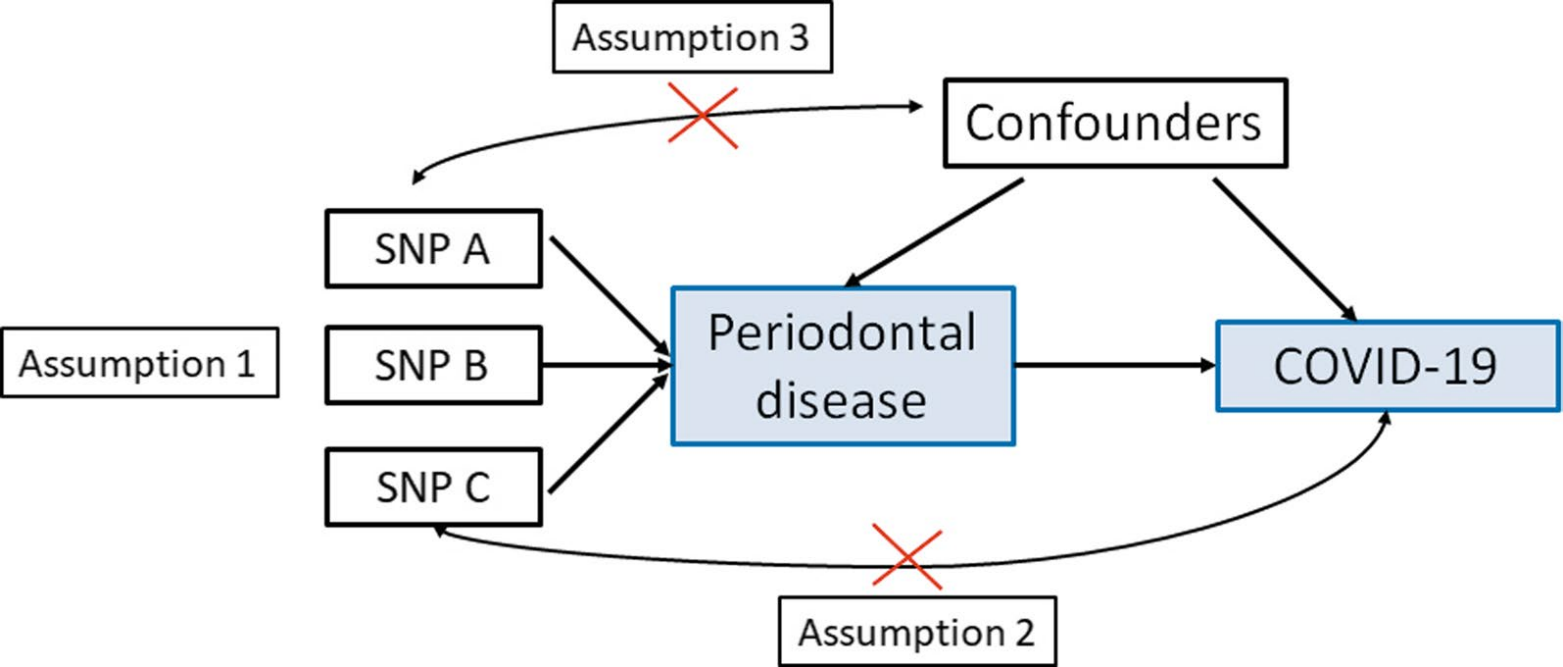
Principles of MR and the assumptions required to obtain an unbiased causal effect estimate

This study is a 2-sample MR design, which allows estimating the causal influence of the exposure on the outcome using GWAS summary statistics from two independent studies. The MR design relies on three assumptions (Fig. 1) [25]. First, the genetic instrument should be strongly correlated with PD. Second, the genetic instruments can only be associated with COVID-19 via PD, also known as exclusion restriction assumption. Third, the genetic instruments cannot be associated with any confounders of the exposure-outcome association.

### GWAS summary statistics for PD

The PD-related trait was searched in MR-Base NHGRI-EBI GWAS Catalog (https://gwas.mrcieu.ac.uk/) for available GWAS summary statistics. Only those datasets that could be directly downloaded into TwoSampleMR R package for MR analysis (https://github.com/MRCIEU/TwoSampleMR) were considered. To satisfy the first assumption, the datasets should contain genetic variants that are identified using the genome-wide levels of statistical significance. Finally, only one available dataset with loci that reached genome-wide levels was identified, i.e., PD-related phenotype (Socransky) [23], comprising genotype data on 975 individuals. The detailed description has been summarized in Table 1.

**Table 1:** Description of the GWAS summary statistics

Trait	GWAS Catalog accession number	Sample size	Number of SNPs	Population	References
Periodontal disease (Socransky phenotype)	ebi-a-GCST003484	975	2,077,804	European	[23]
COVID-19 susceptibility (COVID-19 vs. population)	ebi-a-GCST010776	1,299,010	11,435,708	European	[24]
COVID-19 severity (hospitalized vs. population)	ebi-a-GCST010777	908,494	12,832,272	European	[24]
COVID-19 severity (very severe respiratory confirmed vs. not hospitalized)	ebi-a-GCST010775	957	9,201,012	European	[24]

### GWAS summary statistics for COVID-19 disease

The COVID-19 datasets were from COVID-19 Host Genetics Initiative (https://www.covid19hg.org; Release 4), an international genetics collaboration that aims to discover the genetic determinants of outcomes related to COVID-19 susceptibility and severity [24]. The GWAS results from the comparison of COVID positive versus population controls were used to analyze COVID-19 susceptibility, which comprises 14,134 cases and 1,284,876 controls (Table 1). As for the investigations of COVID-19 severity, cohorts based on two different comparisons were used in this study. The first cohort is based on the comparison of hospitalization (n = 6,406) versus population controls (n = 902,088). The second cohort is from the comparison of very severe respiratory confirmed cases (n = 269) versus not hospitalized COVID-19 (n = 688) (Table 1).

### MR analysis

Analyses were performed using the R package TwoSampleMR v0.5.5 (https://github.com/MRCIEU/TwoSampleMR) [26]. To guarantee that the association with the exposure was robust (Assumption 1), only SNPs with genome-wide significant variants (*P* < 5 × 10^–8^) were included in the MR analysis. Additionally, variants correlated with the most significant SNPs were removed to ensure only LD-independent genetic variants were remained in the analysis (clumping *r*2 cut-off = 0.001 and clumping window = 10,000 kb). Subsequently, all included SNPs were harmonized. It indicates that the effect of a SNP on the exposure and the effect of that SNP on the outcome must each correspond to the same allele. Inverse-variance weighted (IVW) method uses a meta-analysis approach to combine Wald estimates for each SNP, effectively treating each SNP as a valid natural experiment. In the present study, the IVW method was adopted as the main analysis for the estimation on the causal relationships of PD with COVID-19. *P*-Value < 0.05 was considered as the level of significance.

### Sensitivity analysis

Comprehensive sensitivity analyses were performed with the use of TwoSampleMR R package for estimating the robustness of the significant association based on the IVW method (*P* value < 0.05) and potential violations of the MR assumptions (the second and third assumption). Three additional methods were used which allow the existence of horizontal pleiotropy but has lower statistical power than IVW: (1) MR-Egger regression [27]; (2) Weighted median method [28]; (3) Weighted mode method [26]. In addition to these MR methods, the following sensitivity analyses were also applied in this study: (1) Egger intercept calculation [27]; (2) MR pleiotropy residual sum and outlier (MR-PRESSO) test [29]; (3) Heterogeneity tests [30]; (4) Leave-one-out analysis [26].

### Ethical statement

This study only used publicly available data, and the relevant ethical approval can be found in the corresponding studies referenced in the Methods section.

## Results

### MR effect of PD on COVID-19 susceptibility

**Fig. 2:**
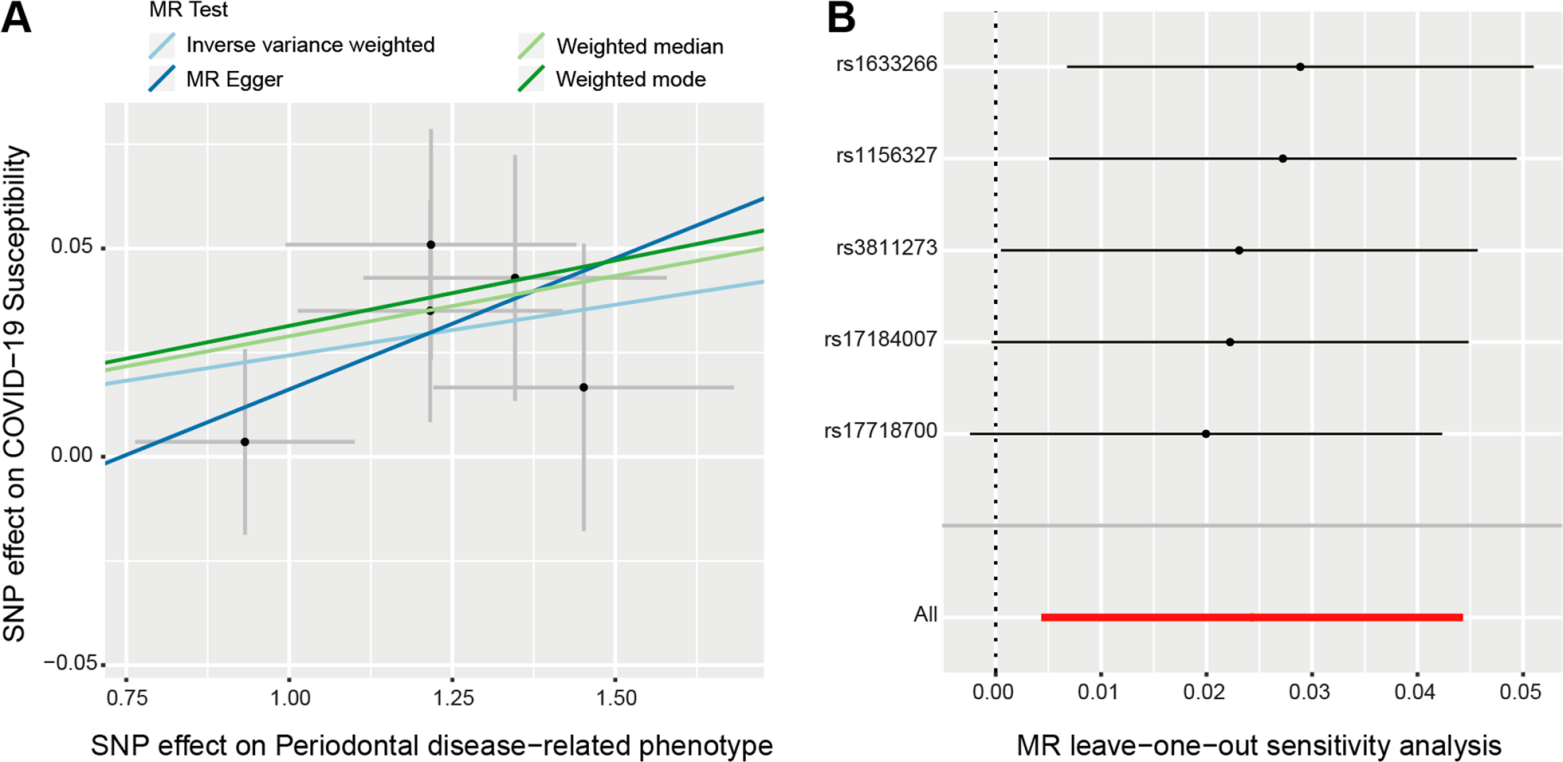
MR analysis and leave-one-out analysis of the causal effect of PD on COVID-19 susceptibility. **A** Scatter plots for MR analyses of the causal effect of PD on COVID-19 susceptibility. The slope of each line corresponding to the estimated MR effect per method. **B** Leave-one-out analysis of the causal effect of PD on COVID-19 susceptibility. Each black point represents the IVW MR method applied to estimate the causal effect of PD on COVID-19 susceptibility excluding that particular variant from the analysis. The red point represents the IVW estimate using all SNPs

Eventually, five SNPs remained for the MR analysis, including rs1156327, rs1633266, rs17184007, rs17718700, and rs3811273. The MR analyses using different methods to estimate the causal inference of PD on COVID-19 susceptibility and severity were presented in Table 2. Interestingly, the MR estimates indicated that PD was significantly associated with COVID-19 susceptibility using the IVW (OR = 1.024, 95% CI 1.004–1.045, *P* = 0.017) and weighted median method (OR = 1.029, 95% CI 1.003–1.055, *P* = 0.024) (Table 2). Such causal association was presented in the scatter plot (Fig. 2A). MR Egger and Weighted mode method also showed the same direction of effect (OR > 1), though the *P* value was not statistically significant. The consistent effects from four different methods suggested that PD could be a risk factor for COVID-19 susceptibility.

**Table 2:** MR results of periodontal disease on risk of COVID-19 susceptibility and severity

Outcomes	Methods	OR	(95% CI)	*P* value
COVID-19 susceptibility (COVID-19 vs. population)	Inverse variance weighted	**1.024**	**1.004–1.045**	**0.017**
	MR Egger	1.064	0.933–1.214	0.418
	Weighted median	**1.029**	**1.003–1.055**	**0.024**
	Weighted mode	1.031	0.998–1.066	0.135
COVID-19 severity (Hospitalized vs. population)	Inverse variance weighted	**1.025**	**1.001–1.049**	**0.039**
	MR Egger	1.053	0.893–1.240	0.580
	Weighted median	**1.030**	**1.003–1.058**	**0.027**
	Weighted mode	1.046	0.999–1.094	0.139
COVID-19 severity (Very severe respiratory confirmed vs. not hospitalized)	Inverse variance weighted	0.964	0.797–1.166	0.711
	MR Egger	1.305	0.356–4.784	0.713
	Weighted median	1.004	0.784–1.287	0.967
	Weighted mode	1.087	0.789–1.499	0.652

*OR* odds ratio, *95% CI* 95% confidence interval

Significant associations (*P* value < 0.05) are highlighted in bold format

Comprehensive sensitivity analyses were then performed to assess the robustness of the causal relationship between PD and COVID-19 susceptibility. An Egger intercept close to zero with *P* value > 0.05 suggests there is no directional horizontal pleiotropy. Thus, no evidence of directional horizontal pleiotropy effects was observed using MR Egger intercept test (intercept = − 0.032, *P* = 0.76). Second, results from MR-PRESSO test also showed there were no horizontal pleiotropic outliers to distort our result (Global Test *P*-value = 0.304). Additionally, heterogeneity test also suggested that no significant horizontal pleiotropy and heterogeneities (*P* value of IVW method: 0.26; *P* value of MR Egger method: 0.16). Furthermore, there were no outliers among these included 5 SNPs based on leave-one-out analysis. The plot of leave-one-out analysis is shown in Fig. 2B. Taken together, the evidence further supported the causal role of PD on the host susceptibility to COVID-19.

### MR effect of PD on COVID-19 severity

**Fig. 3:**
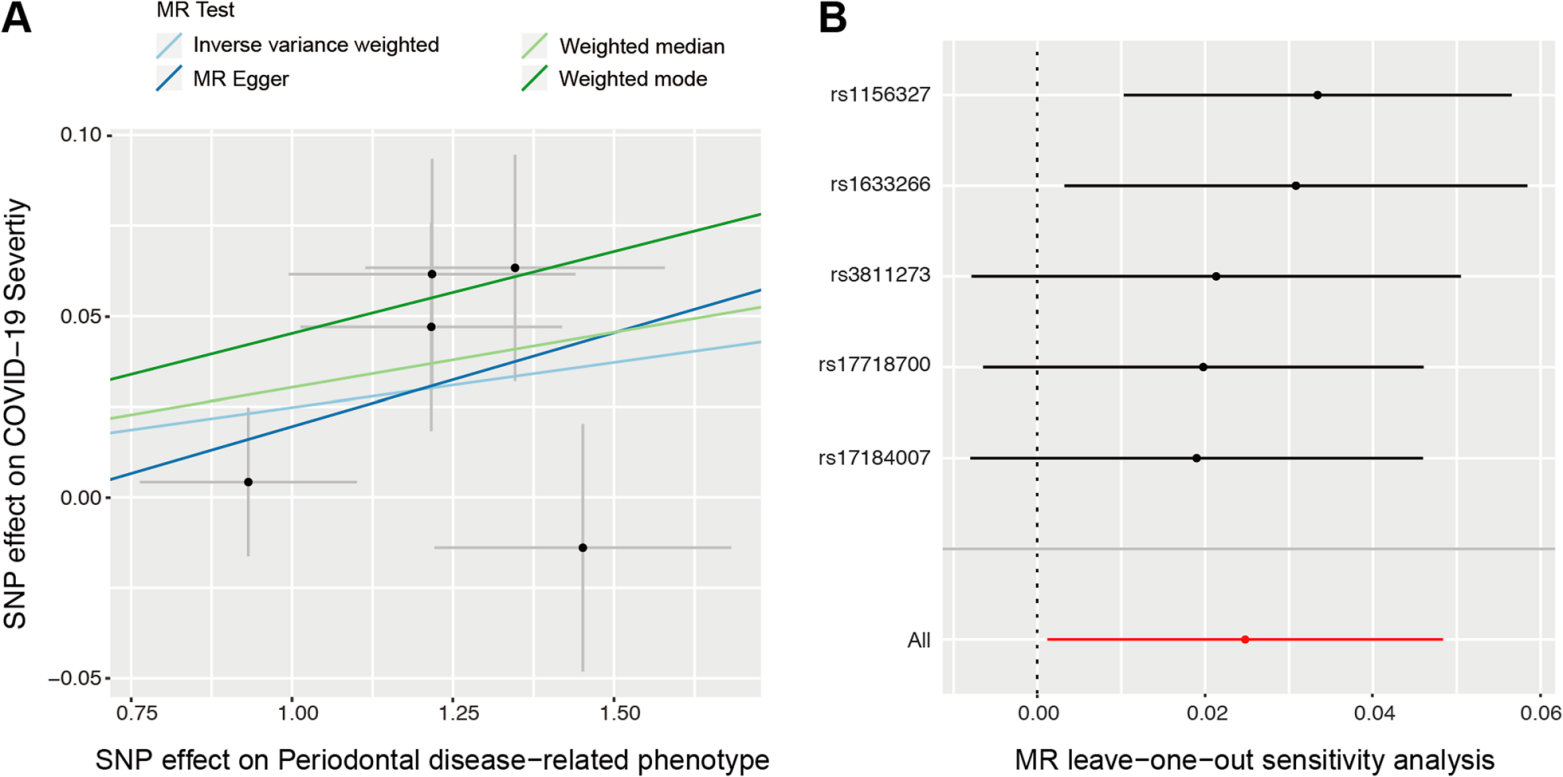
MR analysis and leave-one-out analysis of the causal effect of PD on COVID-19 severity (Hospitalized vs. population). **A** Scatter plots for MR analyses of the causal effect of PD on COVID-19 severity (Hospitalized vs. population). The slope of each line corresponding to the estimated MR effect per method. **B** Leave-one-out analysis of the causal effect of PD on COVID-19 severity (Hospitalized vs. population). Each black point represents the IVW MR method applied to estimate the causal effect of PD on COVID-19 susceptibility excluding that particular variant from the analysis. The red point represents the IVW estimate using all SNPs

Importantly, the MR estimates demonstrated that PD was also significantly correlated with COVID-19 severity, according to the comparison of hospitalization versus population controls (Table 2, Fig. 3A). Both IVW (OR = 1.025, 95% CI 1.001–1.049, *P* = 0.039) and weighted median (OR = 1.030, 95% CI 1.003–1.058, *P* = 0.027) methods showed statistical significance. MR Egger and weighted mode methods showed consistent direction of the effect, but *P* value was not statistically significant. The evidence from comprehensive sensitivity analyses further supported the robustness of the causal association between PD and COVID-19 severity. Egger intercept test indicated that there is no directional horizontal pleiotropy (intercept = − 0.046, *P* = 0.60). Results from MR-PRESSO test showed there were no horizontal pleiotropic outliers to distort our result (Global Test *P*-value = 0.783). Moreover, heterogeneity test also suggested that no significant horizontal pleiotropy and heterogeneities (*P* value of IVW method: 0.77; *P* value of MR Egger method: 0.69). In addition, there were no outliers among these included 5 SNPs based on leave-one-out analysis (Fig. 3B). On the contrary, no association between PD and COVID-19 severity was found from the comparison using the cohort of very severe respiratory confirmed cases versus not hospitalized COVID-19 (Table 2).

## Discussion

MR study provides more credible evidence owing to its resistance to confounding with the use of randomly allocated genetic variants at conception. It has been applied to validate whether these risk factors found in the observational findings (e.g., cardiometabolic glycemic and psychiatric trait) have potentially causal effects on COVID-19 [20–22]. To the best of our knowledge, this is the first MR study to gain the inference about the causal role of PD on the susceptibility and severity of COVID-19. Our results suggest that genetically proxied PD is significantly associated with an increased risk of susceptibility and hospitalization of COVID-19. On the contrary, the causal effect of PD on the severe respiratory complication of COVID-19 is not indicated within our current findings.

### Literature context

Our MR findings extend observational evidence suggesting PD is associated with higher susceptibility and severity of COVID-19. In a recent study by Anand et al. [16] they reported a higher susceptibility to COVID-19 in patients with PD (Gingivitis, OR = 17.65; 95% CI 5.95–52.37; mean clinical attachment loss ≥ 2 mm, OR = 8.46; 95% CI 3.47–20.63; severe periodontitis, OR = 11.75; 95% CI 3.89–35.49). Despite the statistical significance, the wide CIs indicate the low precision of OR, possibly due to the small sample size (n = 150). Moreover, given the infeasibility in case/control selection in the pandemic, the case and control groups were complicated by many confounders (e.g., age, hypertension, diabetes, and smoking). Although many of the confounders were adjusted, the residual of confounding may threaten the validity of the estimated OR. On the contrary, no such association was found in another observational study [14]. In this study by Larvin et al., PD was defined as self-reported poor periodontal health from the UK Biobank cohort. However, these questionaries regarding painful gums, bleeding gums and loose teeth are not clinically accurate enough to measure the disease phenotype [31]. These crude measures could be the results of physiological/pathological factors, such as aging, trauma and previous orthodontic treatment, rather than PD [32].

In a previous study, Marouf et al. compared COVID-19 patients with and without complications and concluded that periodontitis was associated with an increased risk of ICU admission, need for assisted ventilation, and death of COVID-19 patients [15]. In the present study, COVID-19 severity is assessed by hospitalization and severe respiratory complications. The phenotype of hospitalization is based on the comparison between hospitalized COVID-19 individuals and population controls, suggesting that it captures individuals with increased progression into severe symptoms. Therefore, our finding on the association of PD with COVID-19 hospitalization may, at least, in part from the COVID-19 severity. However, the estimated risk for severe respiratory complications in COVID-19 is close to null. Given the small sample size of respiratory confirmed cases (n = 269) versus not hospitalized COVID-19 (n = 688), it is possible that MR analysis of COVID-19 respiratory complications is underpowered to detect the small effect of PD, if any.

A number of MR studies have been performed to infer the causal association of genetically predicted periodontitis with systemic commodities including atherosclerosis [33], hypertension [34], cancer [35], and Alzheimer’s disease [36]. Unexpectedly, most of these hypotheses were not proven as assessed by MR, possibly due to the limited genome-wide significant variants identified for clinically determined periodontitis [37–40]. In the present study, PD was characterized as periodontal disease related-Socransky phenotype, suggesting a high correlation and positive loading with pathogens including *P. gingivalis*, *P. intermedia*, *P. nigrescens*, *T. forsythus*, *T. denticola*, *F. nucleatum* and *C. rectus* [23]. Such colonization pattern highlights the dysbiotic microbial community, which is closely associated with the most common form of periodontitis [41]. Given the heterogenicity of the clinical manifestation and disease expression among patients with periodontitis, our inclusion of SNPs with PD related Socransky phenotype may better reflect the specific risk of microbial dysbiosis, rather than possible shared risk factors of PD and COVID-19.

### Possible mechanisms

Several hypothetical mechanisms may help explain the association of PD with COVID-19 risk as evidenced by MR. Oral epithelial cells that constitute the buccal and subgingival part of the periodontal pocket are highly enriched in SARS-CoV-2 receptors, namely ACE2 and CD147 [13, 42, 43]. And CD147 is found to be increased in periodontitis patients [44]. Since saliva and gingival crevicular fluids have been shown to be the source for SARS-CoV-2 [45], it is possible that the ulcerated epithelium of the periodontal pocket caused by PD, could facilitate the transmission of SARS-CoV-2. In addition, the oral-lung microbiome interactions are well-known factors contributing to many infectious diseases [46]. Notably, a higher level of oral commensal bacteria has been detected in the bronchoalveolar lavage fluid of COVID-19 patients [47]. It is therefore assumed that periodontopathic bacteria could be frequently aspirated in COVID-19 patients, regulating the mucosal immune-inflammatory response while promoting SARS-CoV-2 infection [12, 48, 49]. Furthermore, PD elevates systemic inflammation by the continuous production of pro-inflammatory cytokines and chemokines, which potentially aggravate COVID-19 severity [11, 50].

### Clinical relevance

The results of this study have some clinical implications for COVID-19 risk management and stratification. To the extent that PD is a modifiable factor, the suspension of dental services to control the spread of the virus could have worsened periodontal health during the pandemic, and it should be viewed with caution. Alternatives such as remote oral hygiene instructions are highly recommended to reduce the bacterial load, maintaining oral/periodontal health [51]. And the importance of maintaining rigorous oral hygiene should be emphasized, as having PD could be a predisposition to the adverse complications associated with COVID-19. In addition, we recommend poor oral/periodontal health as a risk factor when stratifying individuals who are at higher risk of COVID-19 infection. Accordingly, dentist/periodontist should warn the patients with poor oral hygiene to undertake more rigorous social distancing or shielding. Importantly, vaccines to these high-risk individuals should be prioritized.

### Limitations

Several limitations should be taken into consideration before the interpretation of the results in this MR study. As the sample size of GWAS for PD was small, the main limitation of this study is that it may be underpowered. Although the statistically significant association of PD with COVID-19 susceptibility and severity were identified by different MR methods, the MR estimates were relatively small (OR = 1.024 for COVID-19 susceptibility, OR = 1.025 for COVID-19 hospitalization). It could be explained by the limited SNPs (n = 5) as genetic instruments, which only account for a small proportion in the phenotypic variance of PD. Future GWAS with a larger sample size could assist the discovery of new associations and validation of those findings from this study. Second, the MR findings only reflect the change in COVID-19 risk due to a genetically predisposed (lifetime) status of PD. In other words, the short-term effect of periodontitis on COVID-19 risk is unknown. Future clinical studies are required to confirm the associations. Additionally, the second cohort (n = 957; very severe respiratory confirmed cases vs. not hospitalized COVID-19) for the investigations of COVID-19 severity was small. Although no association was observed in this cohort, future study based on larger sample size is necessary.

## Conclusions

Within the limitation of the study, we provide genetic evidence that supports the possible causality of periodontal disease accounting for the host susceptibility to COVID-19 and increased hospitalization rate. Our findings emphasize the great importance of disease prevention and oral/periodontal healthcare for general wellbeing during the pandemic and beyond.

## Data Availability

All data generated or analyzed during this study are included in this published article and its additional material.

## Abbreviations

COVID-19: Coronavirus disease 2019
PD: Periodontal disease
MR: Mendelian randomization
GWAS: Genome-wide association studies
IVW: Inverse variance weighted
MR-PRESSO: MR pleiotropy residual sum and outlier
SARS-CoV-2: Severe acute respiratory syndrome coronavirus 2
SNP: Single nucleotide polymorphism
COVID-19 HGI: COVID-19 Host Genetics Initiative
OR: Odds ratio
